# Etiology Analysis and Diagnosis and Treatment Strategy of Traumatic Brain Injury Complicated With Hyponatremia

**DOI:** 10.3389/fsurg.2022.848312

**Published:** 2022-02-21

**Authors:** Jianhua Zhang, Wensheng Dong, Xianghong Dou, Jinjin Wang, Peng Yin, Hui Shi

**Affiliations:** ^1^Department of Neurosurgery, The Affiliated Lianyungang Second People's Hospital of Bengbu Medical College, Lianyungang, China; ^2^Department of Neurology, Donghai County People's Hospital, Lianyungang, China

**Keywords:** traumatic head injury, hyponatremia, cause analysis, diagnosis and treatment strategy, APACHE II

## Abstract

**Objective:**

To explore the etiology and diagnosis and treatment strategy of traumatic brain injury complicated with hyponatremia.

**Methods:**

90 patients with traumatic brain injury admitted to our hospital from December 2019 to December 2020 were retrospectively analyzed and divided into hyponatremic group (50 patients) and non-hyponatremic group (40 patients) according to the patients' concomitant hyponatremia, and the clinical data of the two groups were collected and compared. In addition, patients in the hyponatremia group were divided into a control group and an experimental group of 25 patients each according to their order of admission, with the control group receiving conventional treatment and the experimental group using continuous renal replacement therapy (CRRT). Hemodynamic indices, mortality and serum neuron-specific enolase (NSE) indices before and after treatment were compared between the control and experimental groups. The Glasgow coma scale (GCS) was used to assess the degree of coma before and after the treatment in the two groups, and the patients' disease status was assessed using the Acute Physiological and Chronic Health Evaluation Scoring System (APACHE II).

**Results:**

The etiology of traumatic brain injury complicated with hyponatremia is related to the degree of brain injury, ventricular hemorrhage, cerebral edema, and skull base fracture (*P* < 0.05). After the treatment, the hemodynamic indexes, APACHE II scores, death rate, and NSE levels of the experimental group were significantly lower than those of the control group (*P* < 0.001); The experimental group yielded remarkably higher GAC scores as compared to the control group (*P* < 0.001).

**Conclusion:**

The degree of brain injury, ventricular hemorrhage, cerebral edema, and skull base fracture were considered to be the main factors for traumatic brain injury complicated with hyponatremia. Continuous renal replacement therapy can effectively improve the clinical indicators of the patients with a promising curative effect, which merits promotion and application.

## Background

Traumatic brain injury, as a common disease in neurosurgery, refers to the organic damage to the brain tissue caused by severe head trauma (1, 2), with a rather high disability rate and fatality rate. Clinical manifestations include symptoms such as disturbance of consciousness, dizziness, and headache, the delayed treatment for which may give rise to complications such as permanent dysfunction, amnesia, and epilepsy, jeopardizing patients' life safety and hindering the quality of life (3–5). Hyponatremia, defined as serum sodium below 135 mmol/L, is a common complication after traumatic brain injury and often manifests as cerebral salt-wasting syndrome and inappropriate antidiuretic hormone secretion syndrome, which can lead to serious adverse consequences if not treated promptly (6). After traumatic brain injury, the release of anterior pituitary gland adrenocorticotropic hormone increases due to the stress response, which has a certain impact on sodium excretion, and the application of a large amount of dehydrating drugs after traumatic injury can also lead to low sodium, which may cause impaired nerve cell function, resulting in neurological dysfunction and even disability or death in severe cases (7, 8). In addition, patients with traumatic brain injury are frequently complicated with hyponatremia, which may trigger neurological dysfunction or even death and disability in severe cases due to damages to the patients' nerve cells. It has been found clinically that the understanding of the causes of traumatic brain injury complicated by hyponatremia serves to a better prognosis of patients and drives down mortality (9–11). In addition, the clinical treatment of this disease is mainly gastrointestinal sodium supplementation, which has obvious limitations and poor efficacy (12), so it is also urgent to explore more ideal treatment methods. Continuous renal replacement therapy (CRRT) refers to a group of therapeutic techniques for extracorporeal blood purification, which are now widely used in the treatment of critically ill patients with various non-renal diseases (13). It has the advantages of continuous, slow, isotonic and high-volume solute exchange, hemodynamic stability, removal of medium-molecular substances such as inflammatory mediators, and continuous and stable control of electrolyte and acid-base balance (14). To explore the etiology and diagnosis and treatment strategy of traumatic brain injury complicated with hyponatremia, the clinical data of 90 patients with traumatic brain injury admitted to our hospital from 2019 to 2020 were retrospectively analyzed to dissect the etiology, and of the 90 cases included, 50 cases diagnosed with hyponatremia were identified as research objects. The report is as follows:

## Materials and Methods

### General Information

The clinical data of 90 patients with traumatic brain injury admitted to our hospital from December 2019 to December 2020 were retrospectively analyzed to dissect the etiology. Of the 90 cases included, the patients were divided into a hyponatremic group (50 cases) and a non-hyponatremic group (40 cases) according to their concomitant hyponatremia. Clinical data such as gender, age, body mass index (BMI), vasodilator drug application, place of residence, were collected and compared between the hyponatremic group and the non-hyponatremic group. In addition, patients in the hyponatremia group were divided into a control group and an experimental group of 25 patients each according to their order of admission, with the control group receiving conventional treatment and the experimental group using continuous renal replacement therapy.

### Inclusion Criteria

① Patients met the diagnostic criteria (15) for traumatic brain injury; ② Acute hyponatremia occurred within 7 days of onset, and the serum sodium concentration was <125 mmol/L; ③ This study was approved by the hospital ethics committee, and the patients and their families signed the informed consent form after being fully informed of the purpose and process of the study.

### Exclusion Criteria

① With coagulation dysfunction; ② Pregnant and lactating women; ③ Patients with a history of metabolic diseases; ④ Patients with hyponatremia caused by primary diseases such as chronic renal failure and nephrotic syndrome.

### Methods

The control group received conventional treatment. The patient's central venous pressure was closely monitored. After sodium supplementation and intravenous drip of 3% hypertonic fluid, the vascular volume state was evaluated through the patient's clinical indicators, with the fluid intake stabilized at 900 mL each time. The patients were also given an intravenous infusion of 20 mg furosemide injection (manufacturer: Changbaishan Pharmaceutical Co., Ltd.; NMPA Approval Number H22024699; specification 2 ml: 20 mg), 1 time per day. On this basis, patients were supplemented with hypertonic saline, with the rate of serum sodium increase at 9 mmol/L every 24 h to avoid the occurrence of central pontine myelinolysis.

The experimental group was treated with continuous renal replacement therapy. A double-lumen catheter was inserted into the femoral vein to construct temporary vascular access, and a hemofiltration machine (manufacturer: Guangzhou Guolun Technology Co., Ltd.; model: Fresenius-5008S) was used to implement continuous venous hemodiafiltration treatment. During the treatment, the patient's blood Na^+^ concentration was closely monitored for 8 h each time, and the sodium ion concentration of the replacement solution was adjusted according to the actual situation of the patient's blood Na^+^ concentration. On this basis, sodium supplementation was performed according to the patient's blood Na^+^ concentration. After 24 h, the therapy was continued if the patient's blood Na^+^ concentration was still inadequate, and would be terminated when the patient's 72 h blood Na^+^ concentration was maintained at 140 mmol/L−145 mmol/ L.

**Figure 1:**
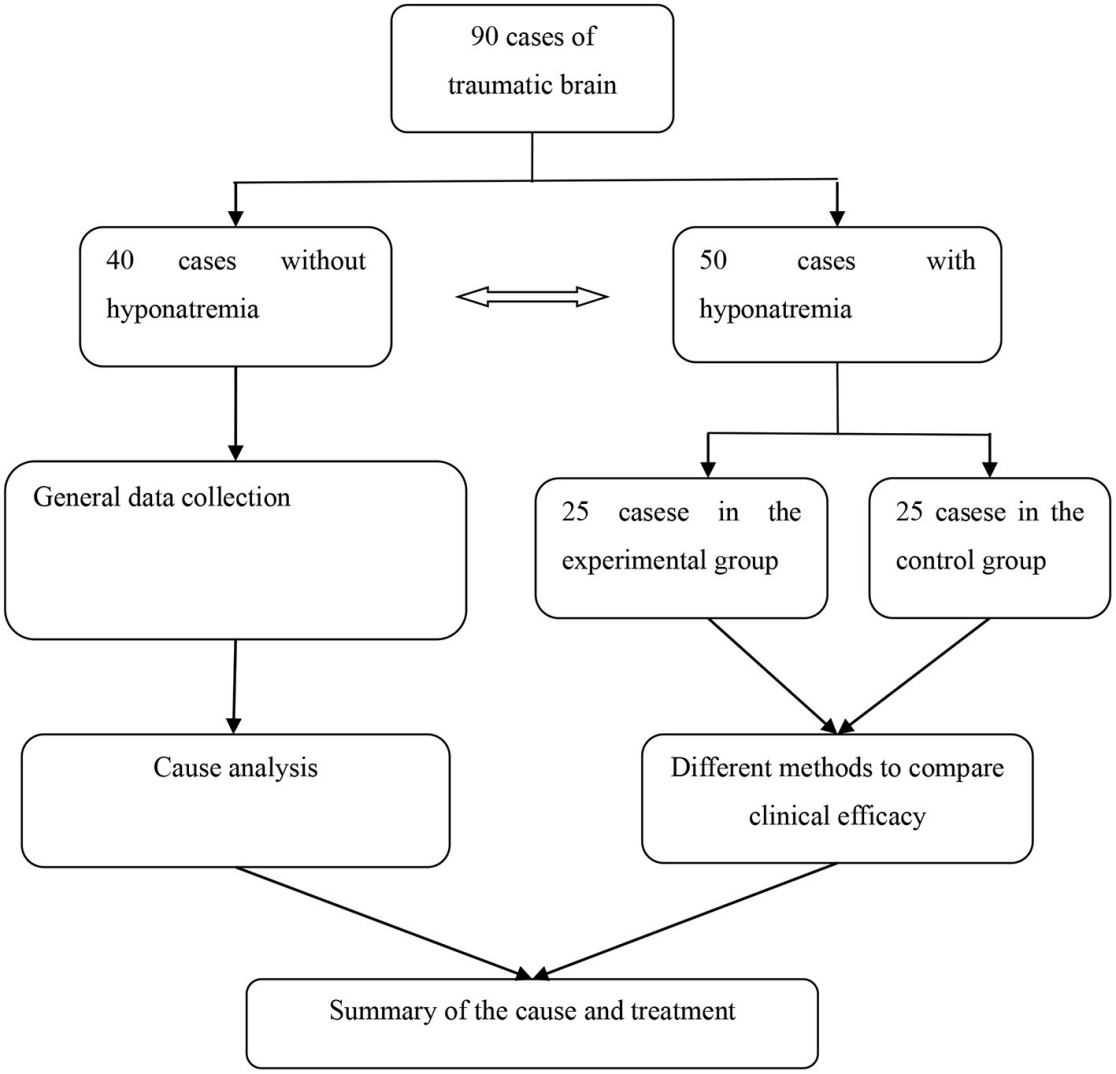
Research flow chart.

All the above treatments were completed in our hospital, and the detailed process was shown in Figure 1.

### Observational Indexes

According to the diagnosis, 90 patients were divided into the non-hyponatremia group and hyponatremia group. Past medical history, degree of traumatic brain injury, mannitol use, clinical manifestations (concussion, ventricular hemorrhage, cerebral edema, and skull base fracture) were collected from both groups to analyze the etiology of hyponatremia complicated by traumatic brain injury.

An ECG monitor (manufacturer: Changhai Berry Electronic Technology Co., Ltd.; model: JHY-40) was used to observe and compare the heart rate (HR) and mean arterial pressure (MAP) in hemodynamics before and after the treatment between the two groups.

The “Glasgow Coma Scale (GCS)” (16) was used to evaluate the coma degree of the two groups of patients before and after the treatment. The GCS includes three scoring systems, including best eye opening (maximum 4 points), best verbal response (maximum 5 points) and best motor response (maximum 6 points), with a total score of 3 to 15 points. The lower the score, the more severe the coma of the patients.

The “acute physiology and chronic health evaluation scoring system (APACHE) II Score Scale” (17) was used to evaluate the patient's condition before and after the treatment. The scale includes components of acute physiology, chronic health status and age, the total score of the scale is 71 points. The higher the score, the more severe the patients' condition.

The death rate of the two groups of patients after the treatment were observed and recorded.

The early morning fasting cubital venous blood of the two groups of patients was collected to separate the serum by centrifugation. The supernatant was collected and stored at −80°C. The serum Neuron-specific enolase (NSE) level in the sample was detected according to the enzyme-linked immunosorbent assay (ELISA) kit instructions. The operation strictly followed the kit instructions and operating procedures for standardized inspections.

### Statistical Processing

SPSS 20.0 software was used to analyze the data in this study, and GraphPad Prism 7(GraphPad Software, San Diego, USA) was used to plot the graphics. The research included count data and measurement data. Count data were expressed by n(%) and analyzed by χ^2^ test, and the measurement data were expressed by (Mean, SD), analyzed by *t*-test and normal distribution. *P* < 0.05 indicated statistical significance.

## Results

### Comparison of General Information

There were no significant differences in gender, average age, BMI, application of vasodilator drugs, and place of residence between the two groups of patients (*P* > 0.05). See Table 1 for details.

**Table 1:** Comparison of general information of the two groups of patients [(mean, SD), n (%)].

	**Hyponatremia group (*n =* 50)**	**Non-hyponatremia group (*n =* 40)**	***χ^2^* or t value**	***P* value**
**Gender**				
Male	27 (54.00)	21 (52.50)	0.020	0.887
Female	23 (46.00)	19 (47.50)		
Average age (years)	49.27 ± 2.31	49.31 ± 2.27	0.082	0.935
BMI (kg/m^2^)	21.55 ± 3.42	21.49 ± 3.31	0.084	0.933
**Application of vasodilator drugs**				
Yes	17 (34.00)	20 (50.00)	2.349	0.125
No	33 (66.00)	20 (50.00)		
**Place of residence**				
Urban	30 (60.00)	25 (62.50)	0.058	0.809
Rural	20 (40.00)	15 (37.50)		

### Analysis of the Etiology of Hyponatremia in Patients With Traumatic Brain Injury

The etiology of patients with complicated hyponatremia had no correlation with past medical history, mannitol application, and concussion (P > 0.05), while it was correlated with the degree of craniocerebral injury, intraventricular hemorrhage, cerebral edema, and skull base fracture (*P* < 0.05). See Table 2 for details.

**Table 2:** Etiology analysis of the patients with hyponatremia [n (%)].

	**Hyponatremia group (*n =* 50)**	**Non-hyponatremia group (*n =* 40)**	***χ^2^* value**	**P value**
Past medical history			0.080	0.777
Diabetes	26 (52.00)	22 (55.00)		
Hypetension	24 (48.00)	18 (45.00)		
**Degree of brain injury**				
Mild	5 (10.00)	13 (32.50)	7.031	0.008
Moderate	17 (34.00)	22 (55.00)	3.991	0.046
Severe	28 (56.00)	5 (12.50)	18.107	<0.001
Use of mannitol			0.172	0.679
Yes	45 (90.00)	37 92.50()		
No	5 (10.00)	3 (7.50)		
Concussion			0.057	0.811
Yes	20 (40.00)	17 (42.50)		
No	30 (60.00)	23 (57.50)		
Ventricular hemorrhage			19.306	<0.001
Yes	37 (74.00)	11 (27.50)		
No	13 (26.00)	29 (72.50)		
Brain edema			6.889	0.009
Yes	35 (70.00)	17 (42.50)		
No	15 (30.00)	23 (57.50)		
Skull base fracture			6.255	0.012
Yes	32 (64.00)	15 (37.50)		
No	18 (36.00)	25 (62.50)		

### Comparison of Hemodynamic Indexes Between the Control Group and the Experimental Group Before and After Treatment

After treatment, HR and MAP in both groups decreased significantly compared with those before treatment, and the levels of each index in the experimental group were significantly lower than those in the control group (*P* < 0.05) (Table 3).

**Table 3:** Comparison of hemodynamic indexes between the two groups (mean, SD).

**Groups**	**HR (time/min)**		**MAP (mmHg)**	
	**Before treatment**	**After treatment**	**Before treatment**	**After treatment**
Experimental group (*n =* 25)	95.37 ± 3.55	75.01 ± 2.13	110.27 ± 22.12	81.55 ± 10.51
Control group (*n =* 25)	94.88 ± 3.69	85.99 ± 3.51	110.12 ± 22.45	98.89 ± 16.35
t value	0.478	13.372	0.024	4.461
P value	0.635	<0.001	0.981	<0.001

### Comparison of GCS Scores Between the Control Group and the Experimental Group

**Figure 2:**
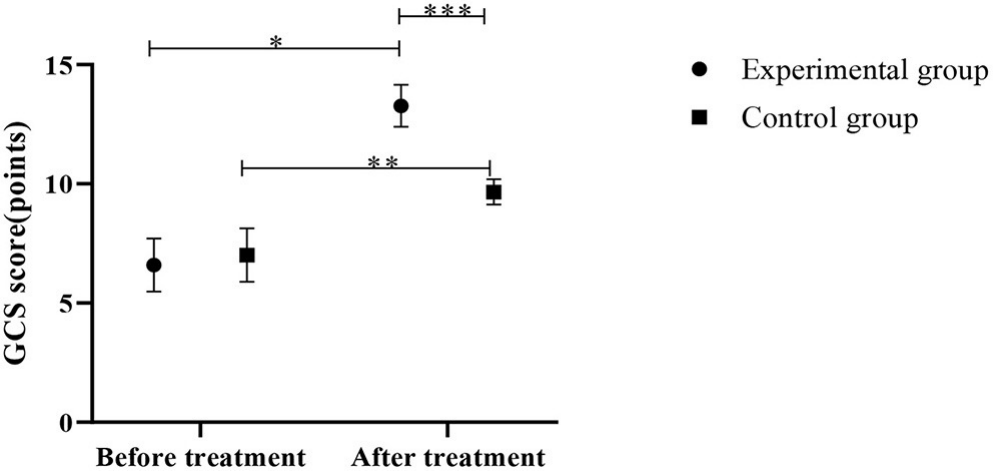
Comparison of GCS scores between the control group and the experimental group (Mean, SD). The abscissa represents before and after the treatment, and the ordinate represents GCS score, points; the GCS scores of patients in the experimental group before and after the treatment were (6.59 ± 1.11) points and (13.27 ± 0.88) points respectively; the GCS scores of the control group before and after the treatment were (7.01 ± 1.12) points and (9.66 ± 0.53) points respectively; ^*^Indicates that there is a significant difference in the GCS scores of the experimental group before and after the treatment (*t* = 23.579, *P* < 0.001); ^**^Indicates that there is a significant difference in the GCS scores of the control group before and after the treatment (*t* = 10.693, *P* < 0.001); ^***^Indicates that there is a significant difference in the GCS scores of the two groups of patients after the treatment (*t* = 17.571, *P* < 0.001).

After treatment, the GCS scores in both groups increased significantly compared to the pre-treatment, strong evidence of significantly higher GCS scores in the experimental group was found, in comparison with those of the control group (*P* < 0.05), as shown in Figure 2.

### Comparison of APACHE II Scores Between the Control Group and the Experimental Group

**Figure 3:**
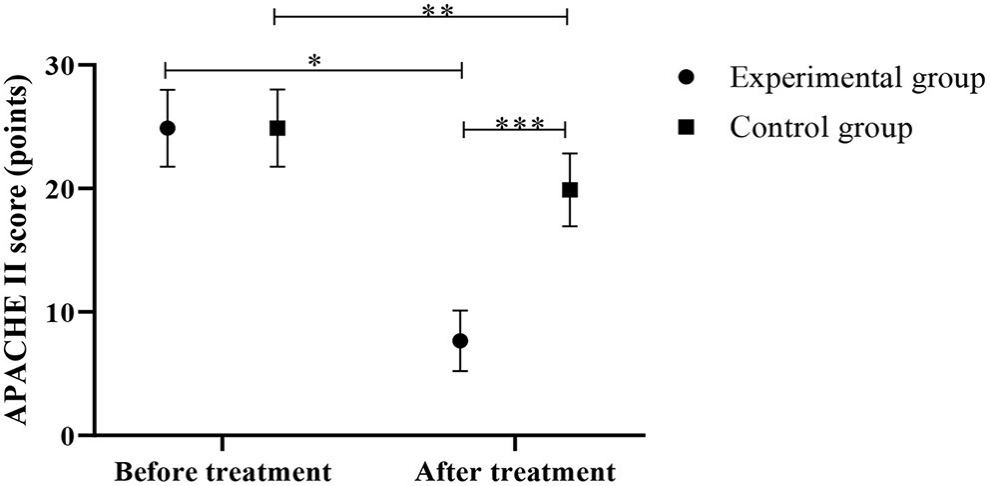
Comparison of APACHE II scores between the control group and the experimental group (Mean, SD). The abscissa represents before and after the treatment, and the ordinate represents APACHE II score, points; the APACHE II scores of patients in the experimental group before and after the treatment were (24.88 ± 3.11) points and (7.65 ± 2.45) points respectively; the APACHE II scores of the control group before and after the treatment were (24.89 ± 3.12) points and (19.88 ± 2.95) points respectively; ^*^Indicates that there is a significant difference in the APACHE II scores of the experimental group before and after the treatment (*t* = 21.759, *P* < 0.001); ^**^Indicates that there is a significant difference in the APACHE II scores of the control group before and after the treatment (*t* = 5.834, *P* < 0.001); ^***^Indicates that there is a significant difference in the APACHE II scores of the two groups of patients after the treatment (*t* = 15.946, *P* < 0.001).

Lower APACHE II scores were observed in the experimental group in contrast to the control group after the treatment (*P* < 0.05) (Figure 3).

### Comparison of Mortality Rate Between Control Group and Experimental Group

**Figure 4:**
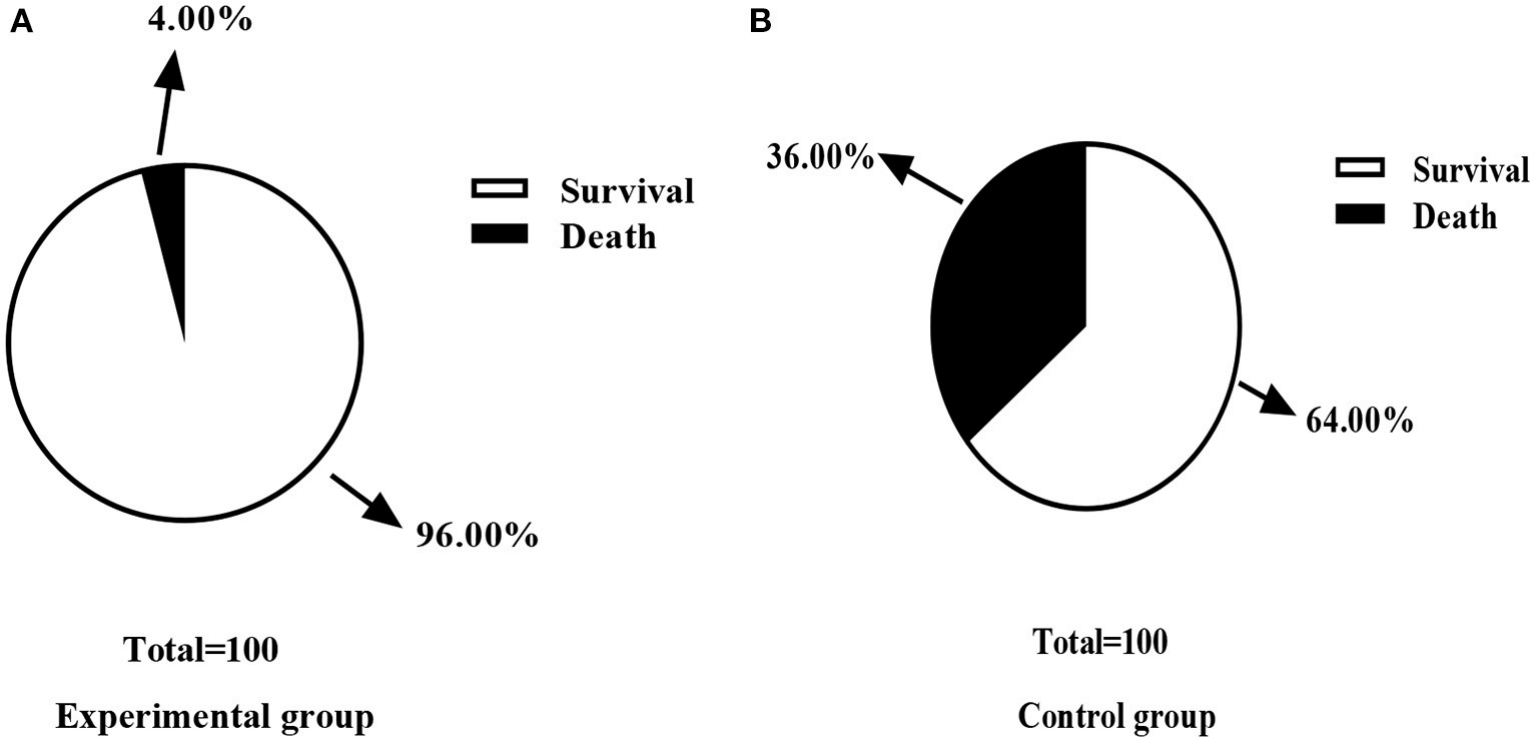
Comparison of mortality rate between control group and experimental group [n (%)]. **(A)** This figure is the death rate of the experimental group; **(B)** is the death rate of the control group; In the experimental group, one case of death (4.00%) and 24 cases of survival (96.00%) were recorded. In the control group, nine cases of death (36.00%) and 16 cases (64.00%) of survival were recorded; There was a significant difference in the death rate between the two groups of patients after the treatment (χ^2^ = 8.000, *P* < 0.05).

There was one case of death in the experimental group and nine cases of death in the control group. The experimental group was recorded with fewer death cases when compared with the control group (*P* < 0.05), as shown in Figure 4.

### Comparison of NSE Levels Between the Control Group and the Experimental Group

**Figure 5:**
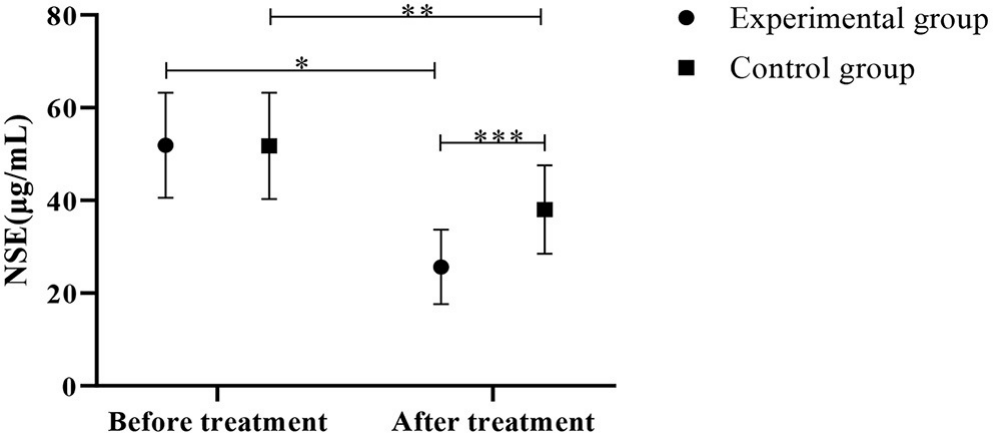
Comparison of NSE levels between the control group and the experimental group (Mean, SD). The abscissa indicates before and after the treatment, and the ordinate indicates the NSE level, μg/mL; The NSE levels of patients in the experimental group before and after the treatment were (51.88 ± 11.33) μg/mL and (25.65 ± 8.02) μg/mL respectively; The NSE levels of patients in the control group before and after the treatment were (51.75 ± 11.45) μg/mL and (38.02 ± 9.52) μg/mL respectively; ^*^Indicates that there is a significant difference in the NSE level of the experimental group before and after the treatment (*t* = 9.448, *P* < 0.001); ^**^Indicates that there is a significant difference in NSE level before and after the treatment in the control group (*t* = 4.610, *P* < 0.001); ^***^Indicates that there is a significant difference in the NSE levels of the two groups of patients after the treatment (*t* = 4.969, *P* < 0.001).

Results in Figure 5 presented lower NSE levels in the experimental group than the control group (*P* < 0.05).

## Discussion

Brain injury is mainly caused by trauma, with hyponatremia as the most influential factor in the prognosis of the disease (18–20). Currently, most scholars believe that the pathogenesis of traumatic brain injury complicated by hyponatremia is related to abnormal secretion of antidiuretic hormone and cerebral salt wasting syndrome (21, 22). Specifically, brain injury predisposes to brain salt depletion syndrome, leading to increased renal sodium excretion and low sodium reabsorption rates, ultimately leading to excessive sodium secretion and hyponatremia (23). In addition, brain injury also stimulates antidiuretic hormone secretion, which strengthens the renal tubule reabsorption capacity, resulting in a significant decline in blood sodium and then hyponatremia. The complications of hyponatremia in traumatic brain injury may induce symptoms such as irritability, nausea, and lethargy, or even death in severe cases if patients fail to receive effective treatment (24, 25). It has been found clinically that the understanding of the etiology of traumatic brain injury complicated by hyponatremia serves to a better prognosis of patients. In this study, it was found that the cause of traumatic brain injury complicated by hyponatremia was related to the degree of brain injury, ventricular hemorrhage, cerebral edema, and skull base fracture (*P* < 0.05), which may be attributed to the following factors (26, 27): ① Severe brain injury inhibits the activity of renal sympathetic nerves, thereby increasing the glomerular filtration and causing the reduction of sodium reabsorption in the renal tubules; ② Abnormal secretion of the antidiuretic hormone leads to hyponatremia as symptoms such as intraventricular hemorrhage and cerebral edema trigger blood circulation disorder, hypothalamus damage, and increased intracranial pressure; ③ Cerebrospinal fluid leakage after skull base fracture impairs the patient's hypothalamic function, resulting in hyponatremia. Clinically, most patients with brain injury are complicated with hyponatremia, which aggravates the condition and increases the mortality rate if effective treatment measures are absent. Therefore, in clinical treatment, after diagnosis of cerebral salt wasting syndrome, sodium supplementation was timely provided to maintain a balanced state of electrolytes in the patient's body. However, the treatment approach is conservative and has limitations (28, 29). CRRT can effectively remove solute and water to restore the electrolyte balance. Furthermore, timely adjustment of the replacement fluid Na^+^ according to the patient's blood Na^+^ concentration avoids the imbalance of blood Na^+^ correction speed and reduces the occurrence of excessive blood pressure osmotic fluctuations. In addition to the correction of hyponatremia, continuous renal replacement therapy effectively relieves cerebral edema, removes nitrogen metabolites, reduces osmotic pressure, and maintains the patient's internal environment in a stable state (30). The results of this experiment showed a significantly lower death rate and a lower NSE level of the experimental group after the treatment than the control group (*P* < 0.05), indicating that the therapy can improve the patient's clinical indicators to reduce mortality. In addition, the present study also found that the GCS score after treatment was higher in the experimental group than in the control group (*P* < 0.05), and the results of James E (31) et al. stated that “the GCS score after treatment was (14.1 ± 0.73) in the treatment group, which was significantly higher than that of (8.9 ± 0.52) in the control group (*P* < 0.05)”, which is generally consistent with the results of this study. It fully indicates that CRRT produced significant clinical effects in improving patients' conditions compared with conventional treatment.

In conclusion, the degree of brain injury, ventricular hemorrhage, cerebral edema, and skull base fracture were considered the main factors for traumatic brain injury complicated with hyponatremia. CRRT can effectively improve the clinical indicators of the patients with a promising curative effect, which merits promotion and application.

## Data Availability Statement

The original contributions presented in the study are included in the article/supplementary material, further inquiries can be directed to the corresponding author.

## Ethics Statement

The studies involving human participants were reviewed and approved by the Ethics Committee of the Affiliated Lianyungang Second People's Hospital of Bengbu Medical College. The patients/participants provided their written informed consent to participate in this study.

## Author Contributions

JZ is responsible for writing the article. WD is responsible for the design of the study. XD is responsible for the collection of case data. JW and PY are responsible for the recording of the results and statistics. HS is the instructor of the entire study. All authors contributed to the article and approved the submitted version.

## Funding

This research is supported by the Lianyungang Municipal Health Commission (202120).

## Conflict of Interest

The authors declare that the research was conducted in the absence of any commercial or financial relationships that could be construed as a potential conflict of interest.

## Publisher's Note

All claims expressed in this article are solely those of the authors and do not necessarily represent those of their affiliated organizations, or those of the publisher, the editors and the reviewers. Any product that may be evaluated in this article, or claim that may be made by its manufacturer, is not guaranteed or endorsed by the publisher.
